# Stomatal Complex Development and F-Actin Organization in Maize Leaf Epidermis Depend on Cellulose Synthesis

**DOI:** 10.3390/molecules23061365

**Published:** 2018-06-06

**Authors:** Emmanuel Panteris, Theonymphi Achlati, Gerasimos Daras, Stamatis Rigas

**Affiliations:** 1 Department of Botany, School of Biology, Aristotle University of Thessaloniki, 54124 Thessaloniki, Greece; fenia_achl@hotmail.com; 2 Department of Biotechnology, Agricultural University of Athens, 11855 Athens, Greece; gdaras@aua.gr

**Keywords:** leaf epidermis, maize, actin, cellulose synthesis, cell wall, stomata, cytoskeleton

## Abstract

Cellulose microfibrils reinforce the cell wall for morphogenesis in plants. Herein, we provide evidence on a series of defects regarding stomatal complex development and F-actin organization in *Zea mays* leaf epidermis, due to inhibition of cellulose synthesis. Formative cell divisions of stomatal complex ontogenesis were delayed or inhibited, resulting in lack of subsidiary cells and frequently in unicellular stomata, with an atypical stomatal pore. Guard cells failed to acquire a dumbbell shape, becoming rounded, while subsidiary cells, whenever present, exhibited aberrant morphogenesis. F-actin organization was also affected, since the stomatal complex-specific arrays were scarcely observed. At late developmental stages, the overall F-actin network was diminished in all epidermal cells, although thick actin bundles persisted. Taken together, stomatal complex development strongly depends on cell wall mechanical properties. Moreover, F-actin organization exhibits a tight relationship with the cell wall.

## 1. Introduction

Plant cells are confined by the cell wall, a rigid container consisting of several polysaccharides, as well as proteins and other materials [1]. The major load-bearing component of the plant cell wall is cellulose, the microfibrils of which regulate cell expansion and morphogenesis by imposing developmental axes, at a single cell, tissue or even organ level [2]. The plant cytoskeleton is considered as the key cytoplasmic organelle that controls cell expansion. Cellulose microfibril orientation is dictated by cortical microtubule organization, as cellulose synthase complexes at the plasma membrane slide along microtubules, while depositing newly-synthesized microfibrils in the cell wall, albeit the presence of microtubules is not required for cellulose synthase movement *per se* [3]. This regulatory effect of cortical microtubule organization on cellulose microfibril patterning is responsible for the shaping of simple cells, such as elongated root epidermal cells, as well as elaborate ones, like lobed mesophyll and ordinary epidermal cells, which are also called “pavement cells” [4].

Furthermore, actin filaments, the second component of plant cytoskeleton, participate in several aspects of plant cell morphogenesis and expansion. In general, F-actin is organized in two distinct arrays in plant cells. Fine cortical microfilaments follow the pattern of cortical microtubules, interconnected to them and to cellulose microfibrils by formin bridges [5]. Deeper in the cytoplasm, thick subcortical F-actin bundles are engaged in the elementary function of cytoplasmic streaming, essential for the distribution and motility of the cytoplasm in vacuolated plant cells, while they orchestrate the movement and location of several organelles, such as the nucleus, endoplasmic reticulum membranes and dictyosomes of the Golgi apparatus [6]. In addition, cortical F-actin aggregations line the plasma membrane at sites of local cell bulging, promoting thus the achievement of elaborate cell shapes [4].

In addition to the regulatory effect of cortical microtubules on cell wall mechanical properties, their bidirectional relationship has been well-established, at least for specific cell types [7]. In particular, it has been shown that defects in cellulose synthesis and deposition affect microtubule organization, by inhibiting cell expansion, in elongating root epidermal cells [8,9,10,11,12]. However, while the cell wall—cortical microtubule bidirectional relationship has been analyzed by several studies, the experimental evidence on the possible effect of cell wall defects on actin filament organization remains scarce. Recently, the influence of cell wall defects on F-actin reorganization in *Arabidopsis thaliana* leaf pavement cells has been reported [13], bringing to light this relationship.

Herein, we elucidate the effect of cellulose deficiency on *Zea mays* leaf epidermis development, using confocal microscopy. Given that leaf epidermis exhibits an elaborate stomatal complex patterning and includes the organization of specialized F-actin arrays, it constitutes an especially suitable experimental system [14,15,16]. Cellulose synthesis was inhibited by 2,6-dichlorobenzonitrile (DCB) [17] or isoxaben [18] and F-actin organization was visualized by fluorescently-labelled phalloidin. Due to cellulose deficiency and the concomitant alteration of cell wall mechanical properties, the pattern of stomatal complex cells was severely affected and, furthermore, F-actin organization was aberrant. The results support the vital role of normal cellulose deposition in the development of such elaborate cell patterns, like those of stomatal complexes in *Z. mays* leaves.

## 2. Results

### 2.1. Seedlings Germinated under the Effect of DCB

**Figure 1:**
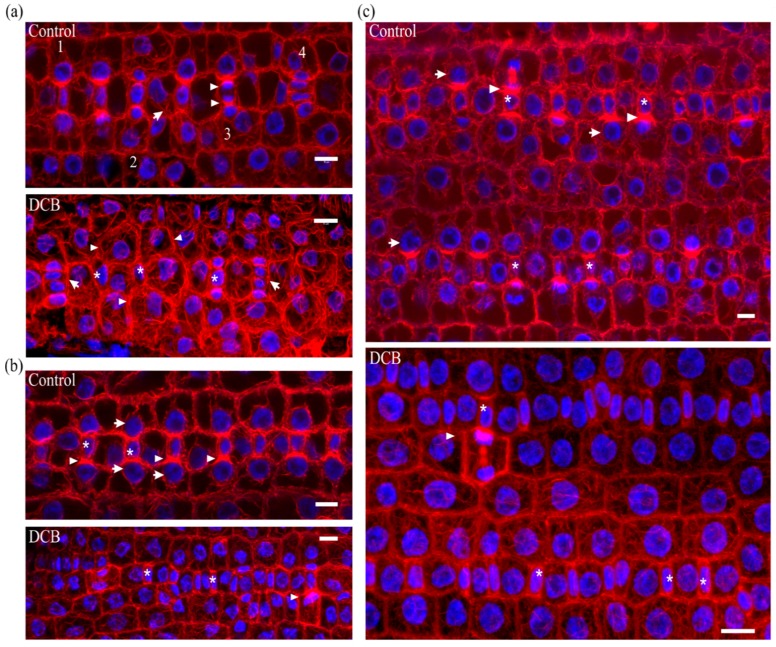
Median CLSM sections of young stomatal rows of control developing *Z. mays* leaves (labeled as “Control”), and maximum projections of serial CLSM sections through protodermal areas of 2 μΜ DCB-germinated *Z. mays* leaves (labeled as “DCB”). F-actin is depicted in red and nuclear DNA in blue. (**a** Control) Progression of stomatal complex ontogenesis, from the stage of initial SMC induction by the GMC (marked as 1), to the stage of subsidiary cell formation (2) and finally to GMC division (3) and fully formed stomatal complex (4). The intervention of a younger stage (arrow) is normal. Note the intense F-actin patches in polarized SMCs and young subsidiary cells, as well as the F-actin aggregations at the pole areas of dividing GMC (arrowheads in 3). (**a** DCB) Abnormal development of a stomatal row. Between two fully formed stomatal complexes (arrows), undivided GMCs (asterisks) and SMCs (arrowheads) can be observed. (**b** Control, **c** Control) Prominent F-actin patches (arrowheads) can be observed at the SMC cortical cytoplasm, at the site of SMC bulging towards the adjacent inducing GMCs (asterisks). Most SMCs appear polarized, as manifested by the juxtaposition of their nuclei to the F-actin patch (arrows). (**b** DCB, **c** DCB) SMCs adjacent to inducing GMCs (asterisks) appear unpolarized, as their nuclei reside away from the inducing GMCs (cf. **b** Control, **c** Control). F-actin patches can be observed only in mitotic/cytokinetic SMCs (arrowheads), while they are absent from SMCs with interphase nuclei. Scale bars: 10 μm.

Stomatal complex ontogenesis in *Zea mays* leaves is accomplished in stomatal rows, by consecutive formative divisions [16]. After the guard cell mother cell (GMC) is generated by an asymmetric transverse cell division, the subsidiary cell mother cells (SMCs), flanking the GMC at either side of the stomatal cell row, are induced to divide asymmetrically to produce a pair of subsidiary cells (stage denoted by 1 in Figure 1a Control). After subsidiary cell production (stage denoted by 2 in Figure 1a Control), the GMC divides symmetrically longitudinally to separate the pair or guard cells (stage denoted by 3 in Figure 1a Control). After accomplishment of all cell divisions, the young stomatal complex consists of 4 cells, two guard cells of the stoma and two subsidiary cells (stage denoted by 4 in Figure 1a Control). Induction of asymmetric SMC division is manifested by polarization of each SMC, the nucleus of which appears anchored adjacent to the inducing GMC (Figure 1b Control, c Control). At the same time, a prominent aggregation of cortical actin filaments, the so-called F-actin patch, is organized under the SMC wall area just beside the inducing GMC (Figure 1b Control, c Control). This F-actin patch persists during SMC division, while it is intensified as SMC bulges towards the inducing GMC and is bequeathed to young subsidiary cells (Figure 1a Control, b Control). Cortical F-actin aggregations also line the longitudinal GMC walls during the symmetric GMC division, over the mitotic spindle pole areas (Figure 1a Control), as is the rule for dividing plant cells [19]. This sequence of ontogenetic events appears more or less continuous, longitudinally to the leaf axis (Figure 1a Control), with GMC formation and induction of asymmetric SMC division closer to the basal leaf meristem, while SMC divisions and GMC division occurring closer to the leaf tip.

In leaves of seedlings germinated in DCB solution (“DCB-germinated” for brevity), stomatal complex ontogenesis exhibited several defects. While GMC formation was generally not affected, SMC polarization and asymmetric division appeared extensively inhibited. In general, few SMCs appeared polarized, as most SMC nuclei were located far from the expected polar site, at the SMC wall area proximal to the inducing GMC (Figure 1b DCB, c DCB; cf. 1b Control, c Control). In addition, F-actin patches were observed only in the SMCs already undergoing division but not to most interphase SMCs (Figure 1b DCB, c DCB; cf. 1b Control, c Control). Furthermore, the continuity of ontogenetic events appeared frequently disrupted, as normally formed stomatal complexes were often alternated with undivided GMCs and/or guard cells with only one subsidiary cell or without any subsidiary cell at all (Figure 1a DCB; cf. 1a Control). Despite the absence of F-actin patches in SMCs, the overall actin filament organization in DCB-germinated leaf protoderm did not appear severely affected at these early ontogenetic stages.

**Figure 2:**
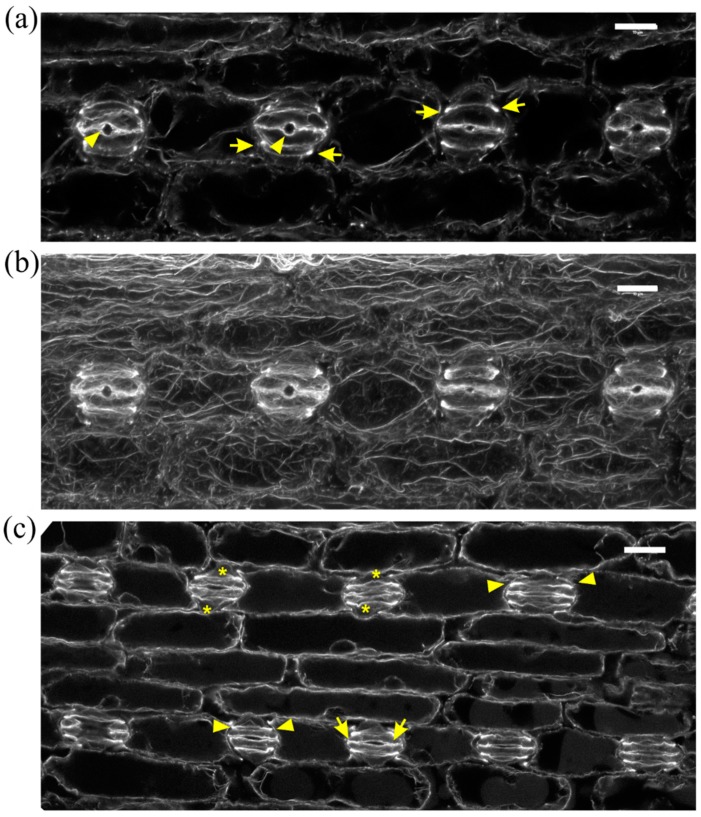
F-actin organization in kidney-shaped (**a**,**b**) and dumbbell-shaped (**c**) stomatal complexes of untreated *Z. mays* leaves. (**a**) Median CLSM section, depicting stomata with kidney-shaped guard cells, exhibiting strong fluorescence along either side of the ventral wall, at the middle of which a circular slot (arrowheads) can be observed. Subsidiary cells are triangular, with intense F-actin aggregations at the lateral junctions with the guard cells (arrows, see also (**b**)). (**b**) Maximum projection of serial CLSM sections of the cells depicted in (**a**), exhibiting a dense network of abundant actin filaments in stomatal complexes and pavement cells. (**c**) Median CLSM section of dumbbell-shaped stomatal complexes, with obtuse-triangular subsidiary cells (asterisks). Intense F-actin signal can be observed at the bulbous ends of ventral guard cell wall (arrows), as well as at the lateral junctions of subsidiary cells with guard cells (arrowheads). Scale bars: 10 μm.

In normally growing *Z. mays* leaves, after formation of stomatal complexes, guard cells gradually become kidney-shaped, with a circular slot at the middle of the ventral wall, while subsidiary cells become triangular at top view (Figure 2a,b). The last step in stomatal complex morphogenesis is the elongation of guard cells until the achievement of dumbbell shape, while the subsidiary cells also elongate to attain an obtuse triangular contour (Figure 2c). During the above stages, intense F-actin fluorescence underlies the stomatal ventral wall, while prominent actin filament aggregations are observed at the lateral junctions of each subsidiary cell wall with the adjacent guard cell (Figure 2a,c). Apart from the above aggregations, an extensive network of fine and thick actin filament bundles traverses throughout the cytoplasm of all epidermal cells (Figure 2b).

**Figure 3:**
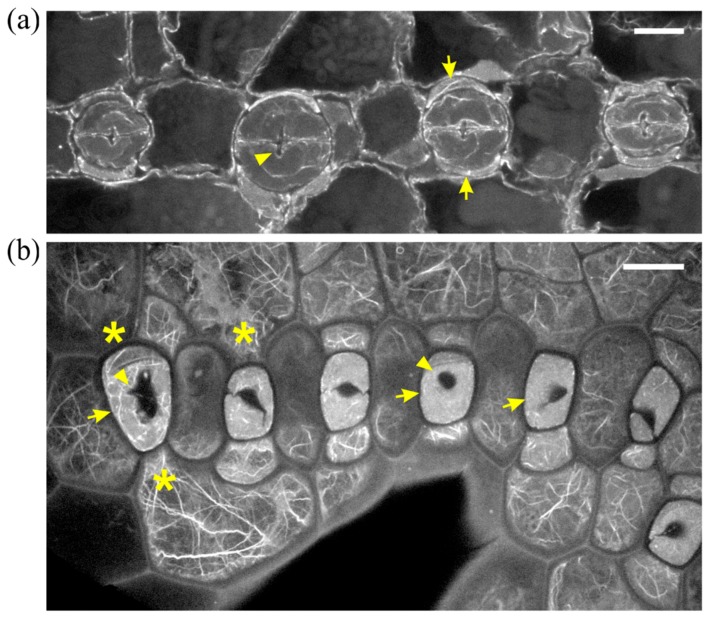
F-actin organization in epidermal areas of 2 μΜ DCB-germinated *Z. mays* leaves. (**a**) Abnormally-rounded stomatal complexes at median CLSM section. Guard cells appear swollen as semi-circles, while a longitudinal slot can be observed vertically to the ventral wall (arrowhead). Subsidiary cells appear crescent-shaped (arrows), compressed between guard cells and pavement cells. None of the F-actin aggregations, typical of this developmental stage, can be observed in these stomatal complexes (cf. Figure 2a,b). (**b**) Severely affected epidermal area, exhibiting highly abnormal stomatal complexes. Several unicellular stomata can be observed (arrows), most of which include an atypical stomatal pore intrusion (arrowheads). Subsidiary cells are abnormally shaped, not exhibiting the typical triangular contour (cf. Figure 2), while in several stomatal complexes there are no subsidiary cells at all (asterisks mark undivided SMCs). None of the F-actin aggregations observed in the untreated kidney- and dumbbell-shaped stomatal complexes (cf. Figure 2) can be observed. Scale bars: 10 μm.

In DCB-germinated leaves, guard cells did not become dumbbell-shaped. In stomatal complexes affected to a lesser extent by the drug, stomata were rounded, with abnormally swollen semi-circular guard cells, forming a longitudinal slot perpendicular to the ventral wall (Figure 3a). Subsidiary cells were crescent-shaped, appearing “compressed” between the swollen stomata and surrounding pavement cells (Figure 3a). In such stomatal complexes, none of the F-actin aggregations of untreated stomatal complexes could be observed (Figure 3a; cf. Figure 2).

**Figure 4:**
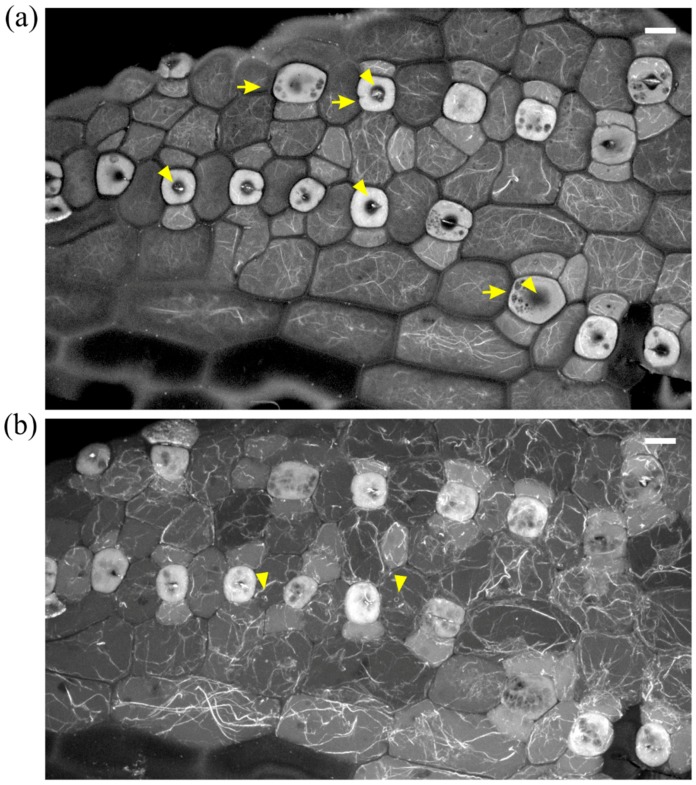
Aberrant cell patterning, abnormal morphogenesis and bundled F-actin network can be observed in this severely affected leaf epidermal area of 2 μΜ DCB-germinated seedlings. (**a**) Single cortical CLSM section; (**b**) maximum projection of serial sections of this area. Unicellular stomata (arrows in (**a**)) with abnormally shaped subsidiary cells and atypical stomatal pore-like intrusions (arrowheads in (**a**)) are abundant. Actin filaments appear diminished, yet bundled and distorted ((**b**); cf. Figure 2b), also exhibiting ring-shaped configurations (arrowheads in (**b**)). Scale bars: 10 μm.

In leaves more severely affected by DCB, stomatal complexes exhibited a variety of aberrations, including malformation or complete lack of subsidiary cells, while stomata were mostly “unicellular”, due to incomplete or totally arrested division of GMCs (Figure 3b and Figure 4). In the majority of these stomata, an atypical stomatal pore was formed, appearing as a “hole” at the external periclinal guard cell wall (Figure 3b and Figure 4a). Similarly to the less affected stomatal complexes, the severely affected ones lacked the F-actin aggregations of untreated stomatal complexes (Figure 3b and Figure 4). In all DCB-germinated samples, the F-actin network appeared diminished at advanced developmental stages, while the existent actin filaments were highly bundled, distorted, often forming rings (Figure 4b, cf. Figure 2b).

### 2.2. Seedlings Treated with Cellulose Biosynthesis Inhibitors after Germination

The defects observed in leaves of DCB-germinated seedlings were also recorded in 3-day-old seedlings treated with cellulose biosynthesis inhibitors namely DCB and isoxaben after germination, albeit to a lesser degree (Table 1). 

**Figure 5:**
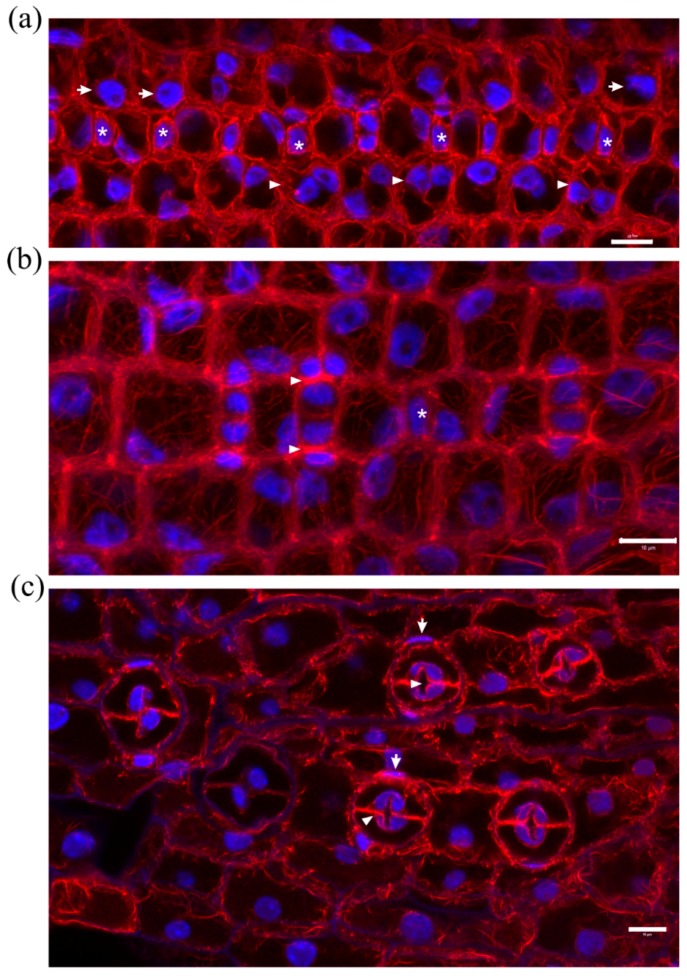
Median CLSM sections of young (**a**,**b**) and mature (**c**) leaf epidermal areas of *Z. mays* seedlings, treated with 2 μΜ DCB after initial germination. F-actin is depicted in red and nuclear DNA in blue. (**a**) SMCs adjacent to inducing GMCs (asterisks) appear unpolarized, as their nuclei (arrows) reside away from the inducing GMCs (cf. Figure 1 Control-labeled images). F-actin patches, like those of untreated SMCs (cf. Figure 1 Control-labeled images) are not observed. In some cases, in which SMC division has produced a young subsidiary cell, the latter appears devoid of nucleus, while the rest SMC includes two nuclei (arrowheads). (**b**) Abnormal development of a stomatal row. Between fully formed stomatal complexes, an undivided GMC (asterisk) flanked by unpolarized SMCs can be observed. One of the young stomatal complexes includes three subsidiary cells (arrowheads). Note that all the young subsidiary cells do not protrude towards the guard cells as in untreated leaves (cf. Figure 1c Control). (**c**) Abnormally-rounded stomatal complexes. Guard cells appear swollen as semi-circles, while a longitudinal slot can be observed vertically to the ventral wall (arrowhead). Subsidiary cells appear crescent-shaped (arrows), compressed between guard cells and pavement cells. None of the F-actin aggregations, typical of this developmental stage, can be observed in these stomatal complexes (cf. Figure 2a,b). Scale bars: 10 μm.

**Figure 6:**
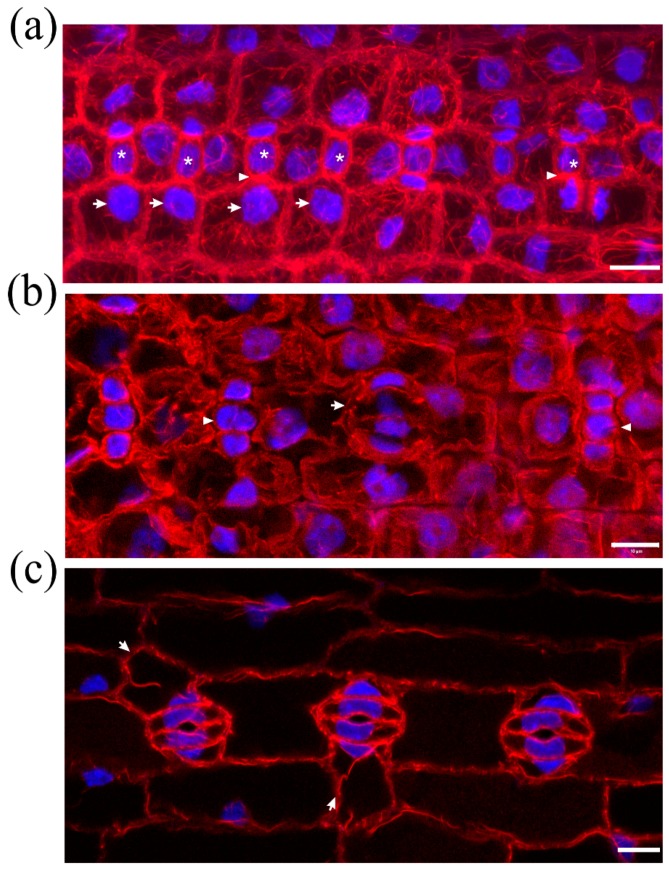
Maximum projections of serial CLSM sections through protodermal leaf areas (**a**,**b**) and single section of mature leaf epidermis (**c**) of *Z. mays* seedlings treated with 10 μΜ isoxaben after germination. F-actin is depicted in red and nuclear DNA in blue. (**a**) SMCs adjacent to inducing GMCs (asterisks) appear unpolarized, as their nuclei (arrows) reside away from the inducing GMCs (cf. Figure 1 Control-labeled images). A prominent F-actin patch, like those of untreated SMCs (cf. Figure 1 Control-labeled images), are observed only in two SMCs (arrowheads), one of which is undergoing cytokinesis. (**b**) An abnormally developed stomatal row, with an almost mature stomatal complex (arrow) between young ones. Among the latter, one with abnormally divided GMC and one with partially divided GMC (arrowheads) can be observed. (**c**) Stomatal complexes with kidney-shaped guard cells. Note the abnormally-shaped subsidiary cells (arrows). Scale bars: 10 μm.

In initiating stomatal complexes, the occurrence of unpolarized SMC nuclei was prominent (Figure 5a and Figure 6a), while F-actin patches were scarce in both SMCs and newly formed subsidiary cells (Figure 5a,b and Figure 6a,b), in contrast to their extensive presence in control leaves (cf. Figure 1 Control-labeled images). 

In addition, several further aberrations were recorded: (a) stomatal rows without developmental continuity (Figure 5b and Figure 6b); (b) young subsidiary cells not protruding towards the stoma (Figure 5b,c) abnormally or partially divided GMCs (Figure 6b).

**Figure 7:**
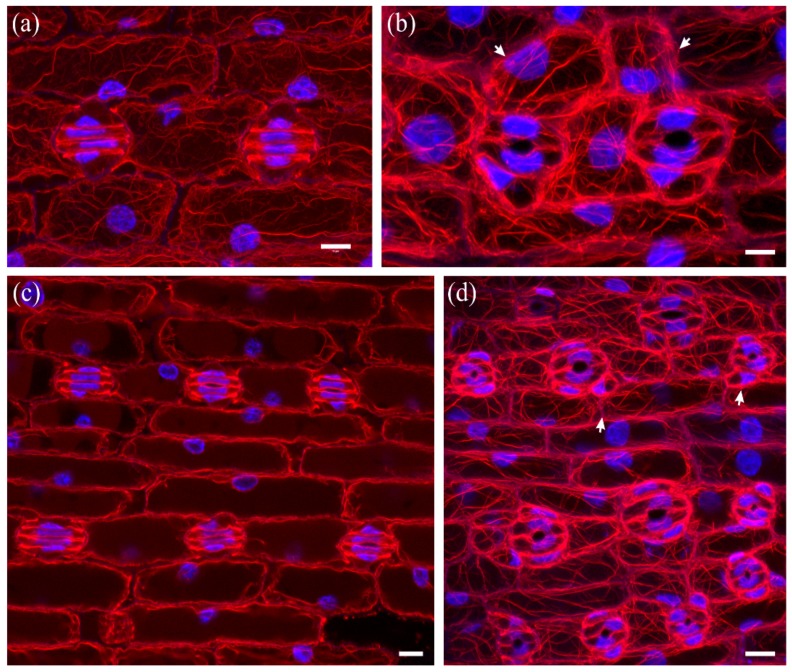
Comparison of developing (**a**) and mature (**c**) untreated stomatal complexes (at single median CLSM section) with 10 μΜ isoxaben-treated mature (**b**,**d**) stomatal complexes (maximum projections of serial CLSM sections). F-actin is depicted in red and nuclear DNA in blue. Note the abnormal swelling of guard cells (**b**,**d**), as compared to the normally elongating ones of untreated leaves (**a**,**c**), and the presence of abnormally-shaped subsidiary cells (arrows in **b**,**d**). In isoxaben-treated epidermis the F-actin aggregations, typical of this developmental stage (cf. Figure 2a,b), are not organized in the stomatal complexes, while extensive F-actin bundling can be also observed in all epidermal cells. Scale bars: 10 μm.

In developed leaf areas reaching maturity, stomata consisted of abnormally swollen semi-circular guard cells, forming a longitudinal slot perpendicular to the ventral wall (Figure 5c), while most subsidiary cells were crescent-shaped, compressed between the swollen stomata and surrounding cells (Figure 5c). Several abnormally shaped subsidiary cells were recorded as well (Figure 6c and Figure 7b,d). Stomatal complexes with elongated (still developing; Figure 7a) or dumbbell-shaped (fully mature; Figure 7c) guard cells were not found in leaves treated with DCB or isoxaben (swollen stomata at maturity; Figure 7b,d), in which the overall cell patterning was affected (Figure 7d), compared with the normal longitudinal cell patterning observed in Figure 7c. In such stomatal complexes, none of the F-actin aggregations of untreated stomatal complexes could be observed (Figure 7b,d; cf. Figure 2), while actin filaments were bundled and misoriented (Figure 7b,d; cf. Figure 2b). 

## 3. Discussion

Here, we show that DCB and isoxaben treatment resulted in inhibition of stomatal complex ontogenesis and malformations during stomatal complex development in *Z. mays* leaves. In addition, F-actin organization in epidermal cells was also affected. While the most severe defects were observed in the leaves of DCB-germinated seedlings, the effects of post-germination treatment with DCB or isoxaben were still evident, but not as frequent as in DCB-germinated seedlings (Table 1). These defects result from the well-appreciated effect of cellulose biosynthesis inhibitors on plant cells [13,17,18]. The expanding cell wall is an armor that, although built and sculpted from the protoplast inside, it also exerts its mechanical properties in an “outgoing” manner, in concert with the expanding walls of neighboring cells, as growing plant cells “push” each other. The significance of mechanical/biophysical signaling for growth and morphogenesis has been underlined, especially for the experimental system of the shoot apical meristem [20,21,22,23]. Besides, the importance of cell wall mechanical integrity has been shown in growing roots, where cellulose shortage results in decreased elongation and reorganization of the cytoskeleton [8,9,10,11,12,24]. Our results reveal the significance of cell wall mechanical strength in a complicated tissue such as leaf epidermis.

While a straight role for F-actin in patterning cellulose microfibrils has only been shown in brown algae [25,26,27], in higher plants actin filaments are involved in cell wall deposition in at least two ways. Firstly, motility of Golgi dictyosomes and vesicles including wall materials is mediated by actomyosin. Inhibition of actomyosin, by either anti-actin or anti-myosin drugs, has been shown to decrease cell growth [11,28,29], probably due to cell wall weakening. The decreased cellulose content and abnormal aggregation of Golgi-derived vesicles, observed in cells of actin mutants, further support this notion [30]. Secondly, cortical actin filaments are directly connected to cellulose microfibrils *via* formin bridges [5]. Likewise, treatment of *A. thaliana* leaves with isoxaben, which inhibits cellulose synthesis and deposition, resulted in decreased F-actin remodeling [13]. Taken together, in dicots there is a tight relationship between actin filament organization and cell wall formation similarly to microtubules [7]. However, the way in which cellulose synthesis inhibitors alter F-actin organization in *Z. mays* stomatal complexes, remains unknown.

In maize leaves affected by cellulose biosynthesis inhibitors like DCB or isoxaben, both SMC polarization/division and F-actin patch organization were affected. As shown by Livanos et al. [31,32], auxin (indole-3-acetic acid) is probably the major inducer of polarization/asymmetric division in SMCs of *Z. mays.* Auxin triggers a cascade of polarizing events [33], including the polar positioning of SMC nucleus aside to the inducing GMC and its further division. It appears that the initiation of these events is based on the special properties of the GMC/SMC wall interface. It is therefore tempting to assume that lack of cellulose, due to DCB or isoxaben, affects the overall cell wall properties, inhibiting thus the induction of SMC division. In addition, the SMC wall area facing the inducing GMC exhibits normally higher expansibility, in comparison to the flanking SMC wall areas [34], resulting in local SMC bulging towards the inducing GMC, which triggers the F-actin patch formation. Consequently, cellulose deficiency due to the inhibitors may affect this expansibility pattern, resulting in inhibition of SMC local bulging. This, in turn, alone or in coordination with failure in auxin transport towards the SMC, prevents the formation of the F-actin patch. Indeed, auxin has been shown to control F-actin reorganization in SMCs [31,32].

Similarly to the F-actin patch of SMCs, the remaining stomatal complex-specific F-actin arrays, observed in untreated leaves, are organized at sites of extra cell bulging, in order to exert a protective effect to the underlying plasma membrane [16]. However, due to cellulose shortage, cell walls swell rather abnormally. Guard cells, instead of elongating to acquire a dumbbell shape, become semi-circular, while subsidiary cells fail to attain their typical triangular shape, as the uniform weakening of cell walls deprives both cell types from any local bulging capability. Consequently, the absence of F-actin aggregations at the subsidiary cell lateral junctions with the guard cells may be a side-effect of altered cell expansion, due to cell wall weakening.

In more severely affected epidermal areas of the DCB-germinated seedlings, even the symmetric GMC division was inhibited. This is not unexpected since DCB has been reported to inhibit cytokinesis in tobacco protoplasts [35]. Interestingly, the “unicellular” stomata that occur, though aberrant in shape, exhibit an atypical stomatal pore. It appears thus that stomatal pore formation is an intrinsic property of stomata, as shown also in caffeine-treated *Z. mays* leaves [36]. F-actin diminishing and bundling in these leaf areas, due to cellulose shortage, may be attributed to a failure in the stabilizing connection of cortical actin filaments with cellulose microfibrils by formin bridges [5]. As cellulose microfibrils become scarce, this connection is compromised, resulting in persistence only of the more rigid F-actin bundles, which also appear distorted. It may be concluded that the F-actin/cell wall continuum is significant for actin filament organization and stability in monocots as it is in dicots [5,37]. Future research should clarify the bidirectional relationship between actin and cellulose in plants by perturbing actin polymerization or patterning and testing the effect on cellulose abundance or organization.

## 4. Materials and Methods

Seeds of *Zea mays* cv. Aris, kindly offered by the Cereal Institute of National Agricultural Research Foundation (Thessaloniki, Greece), were germinated in the dark on filter paper soaked with aqueous solution of 2 μM DCB (Sigma-Aldrich, Steinheim, Germany) prepared from a 10 mM stock solution in dimethylsulfoxide (DMSO), or with aqueous solution of 0.02% DMSO as a control. For treatments after seed germination, seedlings that had been germinated in water (3-day-old) were subsequently treated for five days with 2 μM DCB, as previously described, or with aqueous 10 μΜ isoxaben (Pestanal, Sigma-Aldrich, Seelze, Germany), prepared from a dilution of a 20 mM stock solution in DMSO. This unusually high isoxaben concentration was required as monocots are inherently resistant to numerous cellulose biosynthesis inhibitors [38]. For control, seedlings were treated with 0.02% DMSO (for DCB) or with 0.05% DMSO (for isoxaben). All treatments and procedures were performed at room temperature.

Hand-cut sections from leaves of 5/7-day-old DCB-germinated seedlings and seedlings treated with the inhibitors for 5 days after germination were prepared for F-actin imaging as previously described [15]. In brief, the leaf sections were incubated for 20 min with 300 μΜ MBS (3-maleimidobenzoic acid *N*-hydroxysuccinimide ester) in PEM buffer (50 mM PIPES, 5 mM EGTA, 5 mM MgSO_4_, pH 6.8), to stabilize F-actin, before fixation for 1 h in 4% (*w*/*v*) paraformaldehyde in PEM, with addition of 0.1% Triton-X100 and DyLight 554-phalloidin 1:400 (Cell Signaling, Beverly, MA, USA) as F-actin stabilizer. After rinsing with PEM and extraction with 5% DMSO + 1% Triton-X100 in PBS for 1 h, the sections were incubated for 2 h at 37 °C with DyLight 554-phalloidin 1:40 in PBS. Finally, after DNA counterstaining with DAPI (Molecular Probes, Leiden, The Netherlands) and mounting, the specimens were viewed with a Nikon D-Eclipse C1 confocal laser scanning microscope (CLSM, Nikon Instruments Europe BV, Amsterdam, Netherlands , or with a Zeiss LSM780 CLSM (Carl Zeiss Microimaging GmbH, Jena, Germany) with the appropriate filters. The digital images were acquired according to the manufacturer’s instructions and processed with Adobe Photoshop CS6 (version 13.0) applying only linear settings.

## Figures and Tables

**Table 1:** Effects of cellulose biosynthesis inhibitors on stomatal complex development.

		Control *^a^*	Application of 2 μΜ DCB upon Germination *^a^*	Treatment with 2 μΜ DCB after Germination *^a^*	Treatment with 10 μΜ Isoxaben after Germination *^a^*
*ontogenesis of stomatal complexes*	**SMCs without F-actin patch**	7.55 ± 1.77% (32/420)	24.19 ± 2.14% (92/380) ***	19.65 ± 2.81% (81/410) ***	21.85 ± 1.03% (85/390) ***
	**SMCs with unpolarized nucleus**	2.68 ± 1.03% (11/420)	20.58 ± 1.56% (78/380) ***	17.03 ± 2.33% (70/410) ***	20.56 ± 0.95% (80/390) ***
	**defective divisions of GMCs**	0% (0/350)	16.51 ± 3.35% (68/400) ***	0% (0/420)	6.89 ± 2.24% (26/380) ***
	**non-canonical stomatal rows *^b^***	1.58 ± 0.12% (3/190)	32.51 ± 2.43% (65/200) ***	8.63 ± 0.94% (16/185) ***	20.97 ± 4.68% (42/200) ***
*mature stomatal complexes*	**stomata without subsidiary cells**	0% (0/260)	8.47 ± 1.27% (23/270) ***	5.42 ± 1.06% (15/275) ***	6.91 ± 0.89% (18/260) ***
	**stomata with one subsidiary cell**	5.38 ± 1.59% (14/260)	16.87 ± 2.81% (46/270) ***	9.05 ± 2.86% (25/275) *	10.80 ± 2.96% (28/260) **
	**stomata with aberrant subsidiary cells**	1.91 ± 0.92% (5/260)	23.57 ± 4.14% (64/270) ***	19.01 ± 3.44% (52/275) ***	21.69 ± 3.53% (56/260) ***
	**stomata with swollen guard cells**	0% (0/260)	16.54 ± 4.19% (45/270) ***	14.60 ± 1.59% (40/275) ***	17.85 ± 5.57% (46/260) ***

*^a^* Five biological replicates per experiment were performed. In each experiment, the leaf areas with distorted morphology of six maize seedlings were used to quantify the abnormalities of stomatal complexes. The percentage values are shown as mean ± SD. Text in parentheses represents the number of quantified abnormalities per total number of observations. *^b^* Every row included at least six stomatal complexes. The data were compared performing statistical analysis with the Student’s *t*-test. * *P* < 0.05, ** *P* < 0.01 and *** *P* < 0.001, only *P*-values relating to the control are shown.
